# Psychometric Properties of the Health Literacy Scale Used in the Taiwan Longitudinal Study on Middle-Aged and Older People

**DOI:** 10.3390/healthcare9101391

**Published:** 2021-10-18

**Authors:** Ya-Ling Shih, Chia-Jung Hsieh, Pei-Shan Li, Chieh-Yu Liu

**Affiliations:** 1 School of Nursing, College of Nursing, National Taipei University of Nursing and Health Sciences, Taipei 112303, Taiwan; tiffanysyl0902@gmail.com (Y.-L.S.); phli2@vghtpe.gov.tw (P.-S.L.); 2 Department of Nursing, Taipei Veterans General Hospital, Taipei 112303, Taiwan; 3 Department of Speech Language Pathology and Audiology, National Taipei University of Nursing and Health Sciences, Taipei 112303, Taiwan; chiehyu@ntunhs.edu.tw

**Keywords:** health literacy, health literacy scale, reliability, validation

## Abstract

Health literacy, an important factor in public and personal health, is regarded as the core of patient-centered care. Older people with high health literacy are more likely to maintain a healthier lifestyle, with good control and management of chronic diseases, than those lacking or with poor health literacy. **Purpose:** The present study investigated the validity and reliability of the Taiwan Longitudinal Study on Aging (TLSA) Health Literacy Scale. We also evaluated the health literacy of middle-aged and older Taiwanese adults, and its probable association with health outcomes and life satisfaction. **Method:** We analyzed the internal consistency reliability of the nine items of the 2015 TLSA Health Literacy Scale, and their relationship with the demographic variables. Brody Instrumental Activities of Daily Living (IADL) and the Life Satisfaction Index were used for criterion validity. Moreover, exploratory factor analysis was used to examine the construct validity and to test the known-group validity. **Results:** The TLSA health literacy scale has good internal consistency reliability. Criterion-related validity was supported by the fact that the health literacy score was significantly correlated with the IADL and Life Satisfaction Index. Factor analysis indicated a three-factor structure. Known-group validity was supported by the results, showing that middle-aged and older people with good self-reported health status had better health literacy. **Conclusions:** The TLSA health literacy scale is a reliable and valid instrument for measuring health literacy in middle-aged and older people.

## 1. Introduction

Health literacy is an important determinant of public and individual health and is seen as a core element of patient-centered care [1]. Improving national health literacy has become a priority policy for many countries worldwide such as the United States, Australia, Austria, Canada, Germany, and China. Before developing policies and strategies for the health literacy of older adults in Taiwan, it is necessary to clarify the role and limitations of health literacy for population health promotion. A recent study used the European Health Literacy Survey Questionnaire (HLS-EU-Q) to survey 412 older people in Taiwan. The results showed that more than 50% of older people had poor health literacy [2]. Sufficient health literacy enables individual participation, facilitates full empowerment, and elicits better health outcomes. Health literacy influences health behaviors, the use of health services, and invariably affects health outcomes and social health costs. Good health literacy enables appropriate interaction with health, medicine, scientific knowledge, and cultural beliefs, thereby facilitating greater autonomy, personal empowerment, and life satisfaction [3]. Therefore, it is important to develop population-based health literacy measures that are first population-relevant, and then internationally comparable and reliable.

### 1.1. Introduction to the Available Health Literacy Scales

Early health literacy scales mainly assessed “writing” and “reading” abilities. These scales evaluated elementary functional literacy such as obtaining, understanding, and understanding health information. More recently developed scales are inclined toward the assessment of (i) advanced interactive literacy, namely, autonomous participation, communication, and interaction; and (ii) advanced critical literacy, entailing effective application of knowledge and informed decision-making to maintain optimal health. All these scales differed based on design (objective vs. subjective), constituent evaluation points, and intended literacy level probe.

#### 1.1.1. Objective Health Literacy Scales

The measurement tools developed in the early stage were mostly objective cognitive tests examining the basic functional level and directly testing health-related literacy skills. The Rapid Estimate of Adult Literacy in Medicine (REALM) assesses the understanding of medical-related nouns and common nouns including 125 words. The REALM also assesses the ability of adult patients to read and speak common medical nouns, name body parts, and articulate disease names in a progressively difficult manner [4]. The Short Assessment of Health Literacy–Spanish and English (SAHL-S&E) is based on the REALM and contains 32 items. It is suitable for screening people with low health literacy [5]. The Test of Functional Health Literacy in Adults (TOFHLA) evaluates the patient’s ability to perform and read health-related topics involving calculations by means of omissions. The TOFHLA has a total of 50 items, and is a 12-min reading comprehension test [6], while its abridged/short form has 17 items and is a 10-min numeracy test [7]. The Newest Vital Sign (NVS) uses an ice cream nutrition label to test mental calculation, comprehension, application, reading, writing, listening, and speaking. The NVS consists of six items in total, and must be completed within 3 min [8]. The advantage of these objective health literacy scales is that the actual cognitive ability of the subject can be measured, however, they are limited by the relatively few number of aspects measured.

#### 1.1.2. Subjective Health Literacy Scales

Self-reported scales, adjudged subjective in nature, are also used to measure health literacy. These subjective tools integrate multiple categories and factors related to health literacy and use multi-faceted health literacy concepts. The European Health Literacy Survey Questionnaire (HLS-EU-Q) [9] assesses health literacy in three domains (health care, disease prevention, and health promotion), based on four modes (accessing, understanding, evaluation, and application of health information), and using 47 items. The Cronbach’s alpha (α) for general health literacy is 0.97. The All Aspects of Health Literacy Scale (AAHLS) developed by Chinn and McCarthy [10] is based on the basic/functional, communicative/interactive, and critical consciousness framework developed by Nutbeam. Comprising 14 items, the AAHLS with a Cronbach’s α of 0.75 evaluates four aspects, namely health use information, communication with medical staff, health information management, and health autonomy. The advantage of these scales is that they can measure multiple aspects of health literacy. However, known problems with self-reported questionnaires include favoring extreme responses, social desirability bias, or consistent selection of the same responses (halo effect) [11].

#### 1.1.3. Taiwan Health Literacy Scale

Translated scales in Taiwan include the NVS [12] and HLS-EU-Q [13,14]. It is probable that translation into Mandarin narrows the interpretation of the health literacy concepts assessed by these tools, and thus may limit its application in health care situations in other Mandarin-speaking countries. Therefore, Su et al. [15] developed a Taiwan Health Literacy Scale (THLS) similar to the REALM comprising 66 items based on the World Health Organization definitions, and consistent with local health issues. The Cronbach’s α is greater than 0.97. Tsai et al. [16] also developed the Mandarin Health Literacy Scale (MHLS) and Short-Form Mandarin Health Literacy Scale (s-MHLS). The MHLS contains 50 questions, 33 of these, test text reading ability and 17 test digital ability. The s-MHLS containing “Outpatient Dialogue” and “Medication Information” question groups has 11 questions; eight address text reading skills, and three focus on digital skills. The Cronbach’s α for the s-MHLS is 0.95, and its half-reliability is 0.91. Furthermore, Wei et al. [17] adopted Sørensen’s Integrated Model of Health Literacy and Nutbeam’s health literacy framework to construct the Multidimensional Health Literacy Questionnaire (MMHLQ), which includes five dimensions, namely, accessing, understanding, appraising, and applying health information, communication, and interaction. The MMHLQ includes 20 self-reported items, was validated using a cohort of 2394 adults with mean age of 46.7 years, achieves a Cronbach’s α of 0.85–0.90, and an overall Cronbach’s α of 0.94. The adaptation indices for the final model was χ^2^ = 539.34 (df: 165, *p* < 0.001), comparative fit index (CFI) = 0.953, Tucker—Lewis index (TLI) = 0.946, root mean square error of approximation (RMSEA) = 0.063, and standardized root mean square residual (SRMR) = 0.054.

### 1.2. Research Questions and Purposes

Because health literacy is a complex and evolving structure [10], the applicability, feasibility, and acceptability of health literacy measurement tools are constantly under scrutiny. The goal of screening is to quickly and easily identify individuals who do or do not exhibit certain characteristics [18] such as health literacy. However, some scales can take up to half an hour to complete (e.g., the TOFHLA takes at least 22 min). The NVS, developed in the United States, may not be universally applicable to other countries/regions because it is based on a nutrition label for ice cream [11]. The shortcomings of the REALM include its inability to test the patient’s comprehension and counting, and its lack of discrimination of adults with education above the high school level from those without. Scales translated into Mandarin encounter language barriers associated with the Chinese cultural process, especially as health literacy measures need to reflect local health priorities and belief systems. Moreover, the non-Chinese scales mainly target young and middle-aged adults, and the questionnaire length may not be appropriate for an older population.

To address the aforementioned challenges, The Taiwan Longitudinal Study on Aging (TLSA), is based on a nationwide prospective cohort study of a representative randomized sample of middle-aged and older adults. Thus, the collected data are more representative and better reflect the health and living conditions of the characteristic middle-aged and older Taiwanese population, and are more suited for relevant research or serve as a thematic basis for discussions on important national issues such as Taiwan’s aging population, elderly health care issues, and the formulation of welfare policies. In 2015, TLSA began to add a survey of health literacy, using a short-form scale developed by experts. The purpose of this present study investigated whether the TLSA Health Literacy Scale effectively evaluates the health literacy of middle-aged and older people. Using the TLSA survey data of 2015, we analyzed the psychometric properties of the TLSA scale scores including the internal consistency reliability, known group validity, and criterion-related validity as well as assessed its factor structure by exploratory factor analysis to determine the applicability of the TLSA scale. Moreover, cognizant of the role insufficient health literacy plays in the reduced mobility, poor health, often pessimistic outlook, and dissatisfaction with life of the older population, the present study also used the Instrumental Activities of Daily Living (IADL) and Life Satisfaction Index for ‘criterion validity’, and self-rated health status was used for ‘known-group validity’.

## 2. Methods

### 2.1. Study Design and Data Collection

To understand the health and living conditions of middle-aged and older people over the age of 50, Taiwan has been conducting surveys and research known as the “Taiwan Longitudinal Study on Aging” (TLSA) since 1987. Eight sessions were completed between 1989 and 2015 [19]. The TLSA survey adopts stratified random sampling, so the collected data should fully reflect the physical, psychological, and social aspects of the Taiwanese participants. The relevant research results can also serve as an empirical basis for social and health care policy for older people. This study used old generation sample data from the 2015 Long-Term Tracking Survey on the Physical, Mental, and Social Life of the Middle-aged and Elderly in Taiwan (n = 2667). All personal identification information in the TLSA data were encrypted to protect the participants. This study was approved by Fu Jen Catholic University (FJU-IRB No: C109147), and conducted following the Declaration of Helsinki guidelines on research involving human subjects.

### 2.2. Measures

#### 2.2.1. TLSA Health Literacy Scale

TLSA Health Literacy Scale has a total of nine items, which are scored on a 5-point Likert-type scale. The total score range is 9–45. A higher score indicates a worse health literacy.

#### 2.2.2. Instrumental Activities of Daily Living (IADL) Scale

The IADL scale assesses independent living skills. IADL tends to have a greater cognitive component, involve more interaction with one’s environment, and emphasize community activities [20]. The scale includes a total of nine items: “shopping for personal items”, “ability to handle finances”, “traveling by car or public transportation”, “doing heavy housework”, “doing light housework”, “using the telephone”, “food preparation”, “medication use”, and “laundry”. The IADL score ranges from 0 to 3, with 0  =  no difficulty, 1  =  a little difficulty, 2  =  great difficulty, and 3  =  inability to perform. The total score range is 0–27, and higher scores indicate lower ability in activities of daily living. The Cronbach’s α is between 0.83 and 0.88 [21]. In this study, the Cronbach’s α of the scale was 0.97.

#### 2.2.3. Life Satisfaction Index

This survey uses part of the Life Satisfaction Index developed by Neugarten et al. [22]. The 10 questions are scored as “yes” (1) or “no” (0). After reverse conversion of negative questions, the total score has a range of 0–10. A higher total score indicates better life satisfaction. The Cronbach’s α is between 0.73 and 0.75 [21].

#### 2.2.4. Self-Rated Health

Self-rated health (or self-assessed health, or self-perceived health) is based on asking individuals to evaluate their health status on a five-point scale. It is measured by posing the following question: “How would you rate your current health status?” Responses are set out on a scale of very good (1), good (2), fair (3), bad (4), and very bad (5). Higher scores indicate worse self-rated health.

### 2.3. Statistical Analysis

To explore the psychometric properties of the TLSA Health Literacy Scale, the reliability and validity of the data from middle-aged and older adults (age: ≥50 years old) in 2015 were analyzed. First, the Cronbach’s α of this scale score was calculated as an indicator of internal consistency reliability. For demographic variables, the relationships between the total score of the scale and each dimension with age, gender, and education level were calculated. Then, the correlations between the total score of the scale and the IADL and Life Satisfaction Index were calculated and the correlation validity of the TLSA Health Literacy score was explored. Exploratory factor analysis (EFA) was used to investigate the factor structure. Parallel analysis and theory were considered when determining the number of factors. The factor loadings were estimated using principal axis factoring (PAF), and the Promax oblique rotations method was used.

We used analysis of variance to test for differences between self-rated health status and health literacy, followed by Bonferroni Adjustment post-hoc tests to uncover differences between the groups. The working hypothesis was that middle-aged and older people had both better health literacy and better self-rated health status.

All statistical analysis were conducted using IBM SPSS Statistics for Windows, Version 26.0 (IBM Corporation, Armonk, NY, USA). *p*-value < 0.05 was considered to be statistically significant.

## 3. Results

### 3.1. Socio-Demographic Status and TLSA Health Literacy Scores of the Participants

The descriptive statistics of the sample are listed in Table 1. Our sample of 2667 adults contained 1365 females (51.20%) and 1302 males (48.80%). The largest groups were those 65–85 years old and above (78.10%) and those with a primary or junior high school level education (76.40%). The total score of the TLSA Health Literacy Scale was correlated with age (*t* = 14.59, *p* = 0.000), gender (*t* = 8.37, *p* = 0.000), and education level (*t* = 25.68, *p* = 0.000). The total scores of the health literacy scale of those who were older, female, and less educated had increasing trends, indicating limited health literacy. Moreover, the TLSA Health Literacy Scale exhibited strong correlation with the IADL (*r* = 0.45, *p* < 0.001), Life Satisfaction Index (*r* =−0.31, *p* < 0.001), and Self-Rated Health Score (*r* = 0.28, *p* < 0.001). The mean score for the TLSA Health Literacy Scale, IADL, Life Satisfaction Index and Self-Rated Health were 16.49 ± 6.40, 4.33 ± 8.18, 7.28 ± 2.33, and 2.89 ± 1.01, respectively.

The average scores of items on the TLSA Health Literacy Scale ranged from 1.61 to 2.33. The minimum total score of the scale was 9.00, the maximum was 44.00, the mean was 16.49, and the standard deviation was 6.40. Most (61.5%) of the subjects had sufficient health literacy. Among the three dimensions, health promotion was slightly inadequate. About 25.5% and 23.3% of middle-aged and older people responded to items 5 and 8 with “vague and do not know”.

### 3.2. Reliability

Internal consistency was represented by the Cronbach’s α of 0.86. Here, it was found that no deletions of items could improve the original internal consistency coefficient, so there was no need to delete any items. 

### 3.3. Validity

#### 3.3.1. Criterion Validity

The total score of the TLSA health literacy scale was related to IADL and the Life Satisfaction Index. The TLSA health literacy scale demonstrated reasonable discriminant validity with the Life Satisfaction Index, *r* = −0.31 (*p* < 0.01), and IADL had reasonable concurrent validity, *r* = 0.45 (*p* < 0.01). The results of criterion validity indicated that the total score of the TLSA health literacy scale should be able to effectively reflect that better IADL and Life Satisfaction Index scores indicated better health literacy in middle-aged and older adults.

#### 3.3.2. Construct Validity

Exploratory factor analysis (EFA) is a common method for testing the validity of psychological tests, and consequently identifying underlying relationships between the measured variables. We adopted factor loadings to estimate the principal axis factoring for analyzing the factor structure and determining the correlation between the factors. The Promax oblique rotations method was used. The high Kaiser–Meyer–Olkin (KMO) measure of sampling adequacy suggested that our sample may benefit from factor analysis (KMO = 0.84). In addition, the significant Barlett’s sphericity test result (*X*^2^ = 11,159.89; *df* = 36; *p* < 0.000) indicated the suitability of the current data for factor analysis. Visual examination of the scree plot revealed that the TLSA health literacy scale was a three-structured scale. This was confirmed by the factor loadings of three components, which varied between 0.58 to 0.92 (Table 2). The factors extracted by factor analysis could explain the proportion of variation of all variables. Taking eigenvalues of 1 or more as the extraction standard, the three factors could explain 44.85%, 7.89%, and 6.83% of the variable variation, respectively, and 59.57% of the total variance. Closer inspection of the item details revealed that (i) Factor 1 consisted of three items from health care; (ii) Factor 2 consisted of four items from health promotion; and (iii) Factor 3 consisted of two items from disease prevention.

Zero-order correlations to examine the inter-correlations among the three factors, without controlling, holding constant, or ‘partialing out’ any of the factors, revealed that all factors were significantly related (*r* = 0.42–0.58, *p* < 0.000). It was also apparent that each factor involved different dimensions of health literacy.

#### 3.3.3. Known-Group Validity

**Figure 1:**
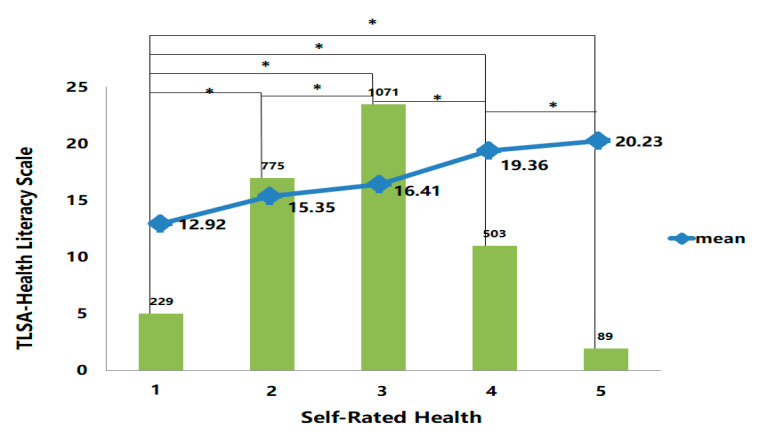
Mean TLSA Health Literacy scale vs. Self-Rated Health. Self-Rated Health: first-very good, n = 229; second group-good, n = 775; third group-fair, n = 1071; fourth group-bad, n = 503; fifth group-very bad, n = 89. TLSA health literacy scale increased with higher self-rated health status (higher scores were associated with worse self-rated health and health literacy). * *p* < 0.05 (Bonferroni corrected *t*-tests).

We examined the analysis of variance (ANOVA) between self-rated health status and health literacy. We found that the five groups of self-rated health status were differentially significant with the subject’s health literacy (*F* = 61.93, *p* = 0.000 < 0.001; Figure 1). As can be seen in Figure 1, on the Bonferroni-corrected *t* test, the group with the best self-rated health status (the first group of the self-rated health status distribution) had the best health literacy score among all groups. Results supported the research hypothesis that middle-aged and older people with higher health literacy were more likely to have good self-reported health status.

## 4. Discussion

### 4.1. Participant Characteristics and the TLSA Health Literacy Scale

The present study found that in terms of gender, middle-aged and older females had lower health literacy scores, which is consistent with reports by Do et al. [23] and Liu et al. [24]. Low education level was also found to be associated with low health literacy scores, as documented in previous research [2,23,24,25,26,27]. We posit that this observation may be related to the historical cultural background of Taiwan, wherein early patriarchy led to inequality in educational opportunity (IEO). Hence, IEO substantially influenced cognitive development, with long-lasting impacts. In particular, women with lower levels of education may have been particularly vulnerable to an educational context of high inequality [28]. 

Consistent with other studies, age was also found to be associated with health literacy, and older age was associated with lower health literacy [2,23,24,25,26,27,29,30]. This can be explained by aging and cognitive decline [31,32]. As individuals advance in age, and their sensory functions possibly deteriorate, this may hinder the ability to receive and utilize information to promote and maintain their own health.

In this study, 61.5% of the middle-aged and older people had sufficient health literacy. This finding is similar to the result of a study in Finland (63.7%) [33], but higher than those in reports from the United Kingdom, 50.5% [25], U.S., 49% [29], Germany, 44% [30], eight EU member states, 41.9% [34], and Turkey, 14.9% [35]. However, this was lower than the figures from Denmark, 82% [26]. This suggests that different countries may have different levels of health literacy due to their different cultural backgrounds.

The inadequate dimension in middle-aged and older people was health promotion, as found in previous studies [2,35,36]. Hence, Uemura et al. [36] suggested that health education through active learning could be effective in enhancing comprehensive health literacy in older adults. On items 5 and 8, about a quarter of the middle-aged and older people responded “vague and do not know”. It is recommended that innovative intervention measures that focus on the health literacy of the older population be developed, tested for efficacy, and implemented. If health literacy is not attained, the consequences for individuals and society could be far-reaching [37]. The negative impact of low health literacy on the health of older adults whose high rate of illnesses require them to have frequent contact with the health care system, follow complicated medical regimes, and make critical medical decisions may be profoundly pronounced [29].

### 4.2. Reliability of the TLSA Health Literacy Scale

In the present study, the TLSA Health Literacy scale was found to have satisfactory internal consistency reliability.

### 4.3. Validity of TLSA Health Literacy Scale 

#### 4.3.1. Criterion Validity

Corollary to McDougall Jr, et al.’s assertion that cognitive and memory decline is related to the decline of IADLs and occurs before the loss of ADLs [38], the present study found that better IADL and Life Satisfaction Index scores were associated with better health literacy in middle-aged and older Taiwanese adults. Our findings indicate that health literacy exhibited the strongest association with the participants’ instrumental activities of daily living, and is consistent with those of Wolf et al. [37] and McDougall et al. [38]. Insufficient health literacy leads to a decline in cognitive skills and reading fluency as well as a gradual decline in understanding of how to stay healthy, when to seek medical care, and how to follow medical plans correctly.

Data presented herein also align with reports that physical factors such as health literacy, health status, functional ability to perform daily basic and instrumental activities, and risk factors are linked to life satisfaction in older adults [39]. In their recently published report, Thapa and Nielsen [27] pointed out that people aged 50–80 years with low health literacy tended to have lower satisfaction with life, forget to take prescribed medicines, and poorly self-assessed their health. Moreover, the health literacy of older individuals is a vital determinant of their life satisfaction and, consequently, quality of life [40].

#### 4.3.2. Construct Validity

Health literacy is a multidimensional concept composed of different factors. The EFA results suggested a three-factor structure of health literacy scale, with 59.57% of the total variance explained. The three factors yielded by EFA align with the health literacy integration model proposed by Sørensen et al. [3]. The core of the model is the ability to access, understand, appraise, and apply health information to make everyday life judgments and decisions that enable a person to navigate the three domains of the health continuum, namely, health care, health promotion, and disease prevention.

#### 4.3.3. Known-Group Validity

Self-rated health is a commonly used measure for subjectively perceived health, and is one of the strongest biological indicators related to death [41]. Some data have suggested that inadequate health literacy is associated with poorer self-rated health status [42]. Our findings are consistent with those of previous studies indicating that health literacy is significantly related to poor self-rated health status [13,24,25,27,29,33,43]. Moreover, Sørensen et al. [34] suggested that the highest proportion of limited health literacy was observed in people who reported poor self-assessed health status.

### 4.4. Limitations

This study has some limitations. First, the individuals in this study were middle-aged and older people living in the community. Compared with individuals living in institutions, they may have had relatively better health literacy. Second, because the health literacy assessment was a component of the first survey in 2015, continuous data analysis could not be performed.

## 5. Conclusions

In conclusion, the presented data indicate that the Taiwan Longitudinal Study on Aging (TLSA) Health Literacy Scale has good psychometric properties such as internal consistency reliability, criterion validity, construct validity, and known-group validity. These results provides a evidential basis and may serve as a reference for scholastic interaction with the TLSA Health Literacy Scale and for future comparison with other health literacy scales. In conclusion, the nine items of the TLSA health literacy scale possess the necessary characteristics including reasonable length, reliability, validity, multi-dimensionality, and a theoretical basis. Therefore, the TLSA scale can be used widely and putatively works as a screening tool, with inherent potential to improve the implementation of health care strategies and policies for older adults with low health literacy. Improved health literacy entails improved health care decision-making, communication, adherence to treatment directions, improved health conditions, and consequently greater patient–provider satisfaction.

It is common knowledge that older people with low health literacy may have lower psychological feelings about themselves, and this often affects their physical and mental health, cause diseases, or aggravates pre-existing disability or impairments. It is thematically-relevant to note that while the present study does not particularly address specific health conditions and/or outcomes that are influenced by health literacy status, there is accruing evidence that low health literacy is strongly associated with mental and age-related chronic systemic diseases such as end stage kidney disease [44], type 2 diabetes [45], asthma, heart failure, obesity, hypertension, arthritis, migraine, prostate cancer stage, acquired immunodeficiency syndrome (AIDS), and depression [46,47]. It is increasingly being understood that improving health literacy has health protective effects, thus, necessitating continued exploration for predictors, protective factors, moderators, and mediators of health/medication literacy. This invariably broadens our understanding of the health literacy of middle-aged and older people, which in turn leads to better health outcomes and successful aging.

## Figures and Tables

**Table 1:** Socio-demographic status and TLSA health literacy scores of the participants.

Variable.	*n*	%	Total		HC		HP		DP	
			*M*	*SD*	*M*	*SD*	*M*	*SD*	*M*	*SD*
Age										
50–64	584	21.90	13.73	4.69	1.27	0.52	1.95	0.88	1.40	0.60
65–85+	2083	78.10	17.26	6.61	1.78	0.87	2.36	1.00	1.54	0.70
Gender										
Male	1302	48.80	15.44	5.87	1.48	0.71	2.18	0.96	1.50	0.70
Female	1365	51.20	17.49	6.73	1.85	0.90	2.35	1.01	1.51	0.66
Education										
Primary or junior high school	2037	76.40	17.80	6.43	1.83	0.87	2.45	0.97	1.56	0.70
High school or university and above	630	23.60	12.25	4.08	1.16	0.41	1.66	0.76	1.32	0.58
HL	2667	100.00	16.49	6.40	1.67	0.83	2.26	0.99	1.51	0.68
IADL	2667	100.00	4.33	8.18	-	-	-	-	-	-
Life Satisfaction Index	2667	100.00	7.28	2.33	-	-	-	-	-	-
Self-rated Health	2667	100.00	2.89	1.01	-	-	-	-	-	-

Abbreviations: M, mean; SD, standard deviation; HC, health care; HP, health promotion; DP, disease prevention; HL, health literacy; IADL, Brody Instrumental Activities of Daily Living Scale.

**Table 2:** Factor analysis and factor loadings of the health literacy scale (n = 2667).

No.	Items	Factor Loading		
		Factor 1	Factor 2	Factor 3
	Eigenvalue	4.41	1.11	1.02
	Variance %	44.85	7.89	6.83
2	When you go to the doctor, can you understand the content or suggestions of the medical staff on the condition or medical treatment?	0.92		
1	When you seek medical treatment, are you able to clearly express or explain your condition to the medical staff serving you?	0.82		
3	Can you read the medication instructions on the medicine bag or understand the medication instructions given by the doctor?	0.74		
9	When you are under pressure, do you know how to find a way to reduce it?		0.79	
8	Did you know that you have to exercise 3 times a week, and each time it takes more than 30 min?		0.74	
5	Can you understand the leaflets or explanatory materials given to you by the hospital on self-control or disease care?		0.71	
7	Will you choose foods that are good for your health?		0.58	
6	Do you usually follow the instructions given to you by the medical staff to control the condition yourself?			0.86
4	Will you follow the medication instructions (such as punctuality and dosage) given to you by your doctor?			0.64

Extraction method: Principal axis factors. Rotation method: Promax method with Kaiser normalization.

## Data Availability

The data that support the findings of this study are available from the Health Data Science Center, Taiwan, but restrictions apply to the availability of these data, which were used under license for the current study, and so are not publicly available. Data are, however, available from the corresponding author upon reasonable request and with the permission of the Taiwan Ministry of Health and Welfare.
